# Why does diseased parathyroid appear weak or heterogenous intensity during intraoperative near-infrared autofluorescence?

**DOI:** 10.3389/fendo.2023.1233610

**Published:** 2023-09-04

**Authors:** Shih-Ming Huang

**Affiliations:** Asian Institute of Thyroid Care, Chang Bing Show Chwan Memorial Hospital, Lukang, Changhua, Taiwan

**Keywords:** diseased parathyroid, hyperparathyroidism, autofluorescence, heterogenous intensity, near-infrared

## Abstract

**Background:**

During intraoperative autofluorescence, the imaging intensity of diseased parathyroid glands is often lower than that of normal parathyroid glands, and some diseased glands especially those in secondary hyperparathyroidism (HPT) show heterogeneous intensities. This study aimed to investigate the reasons for these findings.

**Methods:**

After formalin and paraffin fixation and bivalve cutting, 18 diseased glands from patients with primary HPT, 35 diseased parathyroid glands from patients with uremic HPT, and the surrounding thyroid and thymus tissues were measured using near-infrared autofluorescence with a Fluorobeam imaging system (Fluoptics, France). None of the tissues were stained with indocyanine green. Hematoxylin and eosin staining matched the intensity of the autofluorescence.

**Results:**

Using the bright white intensity of the adult normal parathyroid gland as a reference (index score of 2), the chief cells and oxyphilic cell tissues of the diseased parathyroid had the same intensity score of 2 as that of the normal parathyroid gland, and the clear water parathyroid cell had a weaker intensity score (1–1.5). Their glandular architecture, including the trabecular, follicular, or solid arrangements, did not affect the level of intensity. The thymus, thyroid, fat, fibrosis, and necrosis had very low intensities (scores of 0). The red blood cell-hemorrhage appeared dark black (intensity score -1). The thickness of the fibrotic capsule varied in the diseased parathyroid glands; however, only a very thin capsule was observed in the normal parathyroid glands.

**Conclusions:**

Various degrees of fibrotic capsules in the diseased parathyroid gland may be the main factor contributing to the lower intensity during autofluorescence, and different cell types, necrosis, fibrosis, and hemorrhage may explain the appearance of heterogeneous intensity in the diseased parathyroid glands.

## 1 Introduction

The parathyroid gland has an intrinsic endogenous autofluorescence, emitting a near-infrared (NIR) fluorescent signal with a peak near 820 nm, as described initially by Paras (1). The fluorescence intensity of the parathyroid gland has been shown to be greater than the intensities of the thyroid gland and other neck tissues (1). This has been used to identify normal parathyroid glands during thyroidectomy or central lymph node dissection (2–5). However, during intraoperative autofluorescence, the imaging intensity of diseased parathyroid glands is often lower than that of normal parathyroid glands, and in some diseased glands, especially those in secondary hyperparathyroidism (HPT), are heterogeneous in intensity (6–11). Since all the normal parathyroid glands consist of chief cells, the concentration of the chief cells or the presence of calcium-sensing receptors have been considered to be reasons for the varying intensities (12). However, there has been no definite substantiating evidence for this. Therefore, this study aimed to investigate the reasons for these findings.

## 2 Materials and methods

Patients with a confirmed diagnosis of primary HPT or secondary HPT who underwent parathyroidectomy at the Chang Bing Show Chwan Memorial Hospital between January 2021 and August 2022 were included in this study. The studies involving human participants were reviewed and approved by Show Chwan Memorial Hospital IRB. None of the patients were injected with indocyanine green (ICG) during the operation. After formalin and paraffin fixation and bivalve cutting, the excised tissue, including the diseased and normal parathyroid, thyroid, thymus, and surrounding neck soft tissues, was measured using NIR autofluorescence with a Fluorobeam imaging system (Fluoptics 800, France) at a peak near the 820 nm fluorescent signal, positioned 5 cm above the *ex vivo* formalin fixed parathyroid tissue. The autofluorescence intensity was matched to the pathology using hematoxylin and eosin (H&E) staining.

## 3 Results

**Figure 1:**
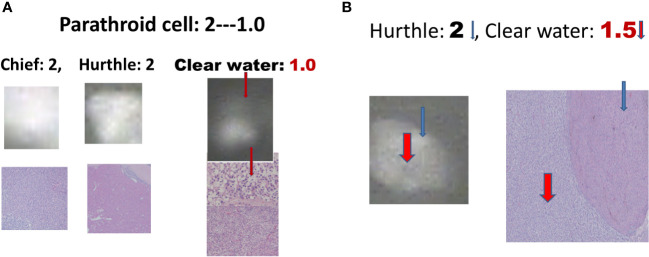
Near-infrared autofluorescence image, and different cell types of parathyroid histology. **(A)** Chief cells, Hurthle cells, and clear water cells (red arrow). **(B)** Hurthle cells (blue arrow), and clear-water cells (red arrow).

**Figure 2:**
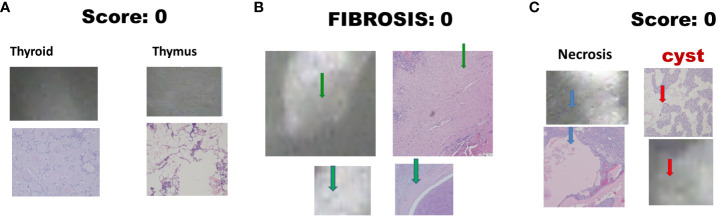
Near-infrared autofluorescence image score 0 and different tissue histology. **(A)** Thyroid, and thymus; **(B)** Fibrosis (green arrow); **(C)** Necrosis (blue arrow), and cystic change (red arrow).

**Figure 3:**
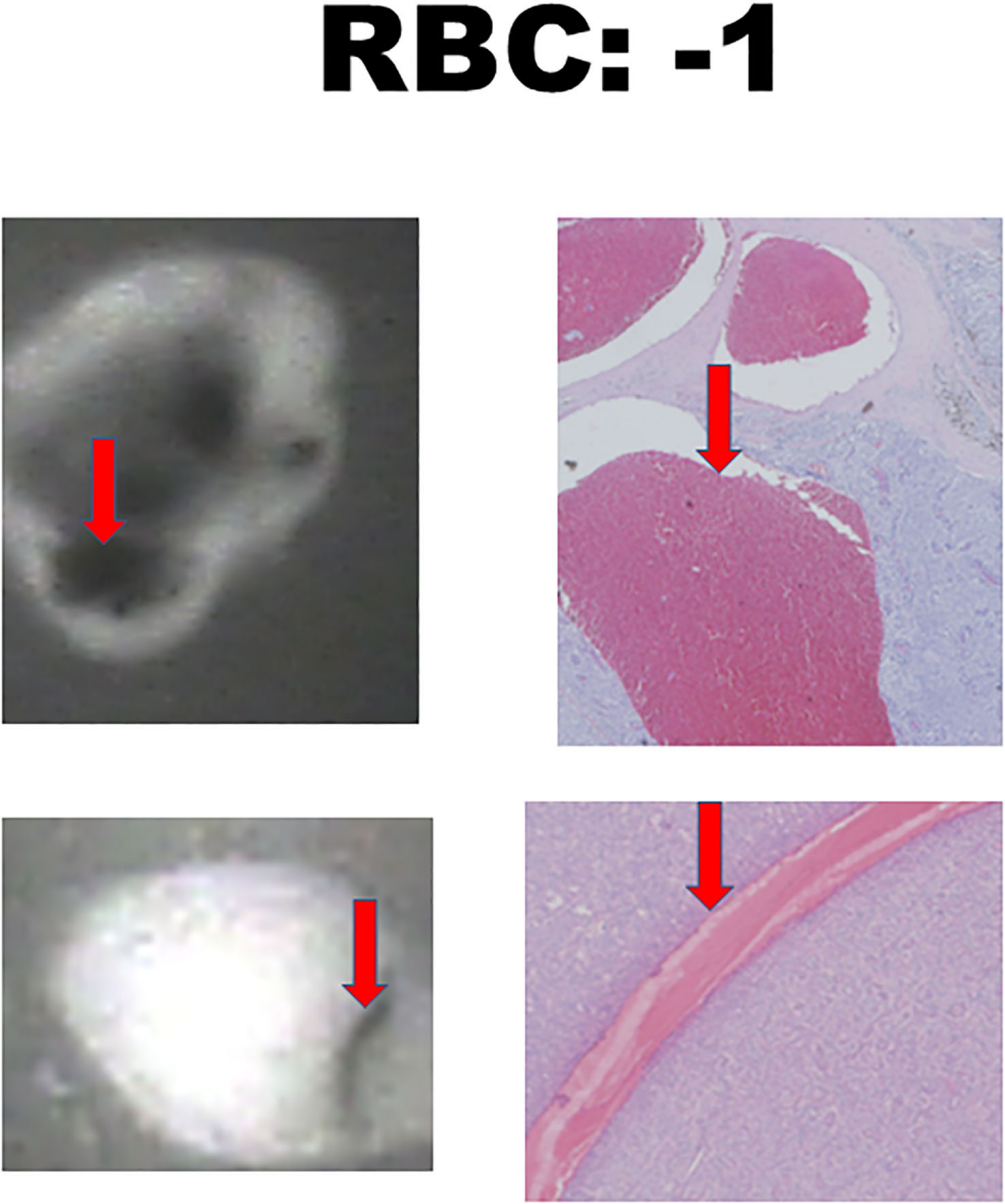
Near-infrared autofluorescence image score -1 and hemorrhage histology. Red blood cells (RBCs) are indicated by the red arrows.

**Figure 4:**
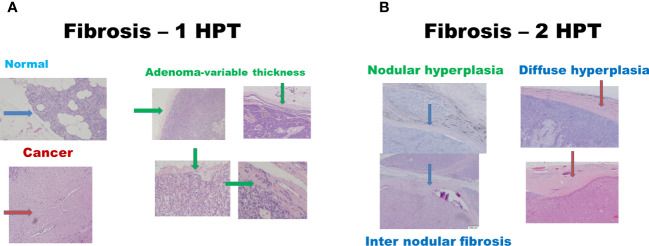
Histologic fibrosis in primary and uremic HPT. **(A)** Primary HPT: normal cells (blue arrow), cancer cells (red arrow), and adenomas (green arrow). **(B)** Uremic HPT: Nodular (blue arrow) and diffuse hyperplasia (red arrow).

There were 17 diseased parathyroid tissues from 12 patients with primary HPT, including three patients with MEN1, eight with single adenomas, one with parathyroid cancer, and six normal parathyroid glands accompanied by diseased tissue. Of the 11 patients with uremic HPT, 35 had a diseased hyperplastic parathyroid gland. Using the bright white intensity of an adult normal parathyroid gland as the reference score 2, the background as score 0, and the darker than background as score -1, the chief cells and oxyphilic cell tissues of the diseased parathyroid had the same intensity score 2 as that of the normal parathyroid gland, and the clear water parathyroid cells appeared to have a weaker white intensity score (1–1.5) (**Figures 1A, B**). The thyroid, thymus (**Figure 2A**), lymphatic tissue, and fat tissue showed background intensity scores of 0. Fibrosis (**Figure 2B**), necrosis, and cystic changes in the parathyroid gland (**Figure 2C**) also showed a background intensity score of 0. The red blood cells appeared very dark (score -1) (**Figure 3**). The glandular architecture, including the trabecular, follicular, and solid arrangements, did not affect the level of intensity. While the thickness of the fibrotic capsule varied in the diseased parathyroid glands, only a very thin capsule was observed in the normal parathyroid glands (**Figure 4A**). The uremic parathyroid gland usually has a thicker fibrotic capsule, and interlobular fibrosis is a key feature of nodular hyperplasia (**Figure 4B**). Strong and thick fibrosis was noted in the capsule and parenchyma of the parathyroid cancer (**Figure 4A**).

## 4 Discussion

The intraoperative intensity depends on the wall thickness and parathyroid tissue content (**Table 1**), with the capsule thickness being the main factor for weak intraoperative intensity. The greatest thickness was observed in parathyroid cancer, followed by uremic parathyroid, primary parathyroid adenoma or hyperplasia, and normal parathyroid tissue. A very thin, one-layer fibrotic capsule of the normal parathyroid gland is the main reason for the normal rim of parathyroid adenoma.

**Table 1:** Summary of the factors affecting the intensity of autofluorescence.

Summation effect:	
1) Wall thickness: weakness
Parathyroid CA> uremic HPT> primary HPT>>>Normal
2) Content: heterogenous
score -1: RBC- hemorrhage
score 0: Necrosis, cyst, fat, and fibrosis
score 1.0-1.5: Clear water cell
score 2: Chief cell, Hurthle cell

CA, carcinoma; HPT, hyperparathyroidism; RBC, red blood cell.

Parathyroid cancer presents typically as a thick capsule. Once bivalve cutting is performed during surgery or after formalin fixation, the intensity becomes stronger, since the wall thickness effect is excluded and the intensity is dependent on the cellular types, area of necrosis, cystic change, fibrosis, or even hemorrhage, which is comparable to the heterogeneous intensity of parathyroid adenoma observed in a previous report (13). Necrosis, cystic change, fibrosis, or even hemorrhage occurred more often in larger parathyroid lesions, which may be either primary or uremic HPT. Intraoperative *ex vivo* bivalve cutting can be used to identify parathyroid gland with the increase in its intensity. Lee et al. reported that smaller parathyroid adenomas had higher near-infrared autofluorescence intensities (9). Our results showed that the thickness of the parathyroid capsule varied from that of the primary HPT. Five of the 16 parathyroid glands of the sixteen primary HPT had a relatively thinner capsule, and four of these five were relatively small adenomas weighing less than 300 mg. Andrea et al. found the absence of NIR autofluorescence in three parathyroid cancers (14). In our case, strong and thick fibrosis was noted in the capsule and parenchyma. Fibrosis, with a score of 0, may have been the reason for this finding.

In uremic HPT, strong inter-septal fibrosis in the nodular hyperplasia also aggravates weakness and heterogeneity. However, the mechanism underlying the autofluorescence remains unclear. Intrinsic biological fluorophores are unaffected by cryopreservation or formalin fixation (15, 16). A calcium-sensing receptor protein was hypothesized to be a fluorophore (12) explaining the decreased intensity of the uremic parathyroid. However, according to our results, the intensity of the chief cells or Hurthle cells in the uremic parathyroid gland was the same as that in the normal parathyroid or parathyroid adenoma, which may be an important aspect for further research — why do clear water cells appear to have a lower intensity than the chief and Hurthle cells?

## Data availability statement

The original contributions presented in the study are included in the article/supplementary material. Further inquiries can be directed to the corresponding author.

## Ethics statement

The studies involving human participants were reviewed and approved by Show Chwan Memorial Hospital. Written informed consent for participation was not required for this study in accordance with the national legislation and the institutional requirements.

## Author contributions

S-MH: Study design, data acquisition and interpretation, and writing and editing of the article.
